# Technology Enabled Clinical Care (TECC): Protocol for a Prospective Longitudinal Cohort Study of Smartphone-Augmented Mental Health Treatment

**DOI:** 10.2196/23771

**Published:** 2021-01-14

**Authors:** Natali Rauseo-Ricupero, John Torous

**Affiliations:** 1 Division of Digital Psychiatry Beth Israel Deaconess Medical Center Harvard Medical School Boston, MA United States

**Keywords:** mental health care, access, multi-language, smartphone, app, quality improvement, protocol, mental health, treatment, acceptability, efficacy, COVID-19

## Abstract

**Background:**

Even before COVID-19, there has been an urgent need to expand access to and quality of mental health care. This paper introduces an 8-week treatment protocol to realize that vision—Technology Enabled Clinical Care (TECC). TECC offers innovation in clinical assessment, monitoring, and interventions for mental health. TECC uses the mindLAMP app to enable digital phenotyping, clinical communication, and smartphone-based exercises that will augment in-person or telehealth virtual visits. TECC exposes participants to an array of evidence-based treatments (cognitive behavioral therapy, dialectical behavior therapy, acceptance and commitment therapy) introduced through clinical sessions and then practiced through interactive activities provided through a smartphone app called mindLAMP.

**Objective:**

TECC will test the feasibility of providing technology-enabled mental health care within an outpatient clinic; explore the practicality for providing this care to individuals with limited English proficiency; and track anxiety, depression, and mood symptoms for participants to measure the effectiveness of the TECC design.

**Methods:**

The TECC study will assess the acceptability and efficacy of this care model in 50 participants as compared to an age- and gender-matched cohort of patients presenting with similar clinical severity of depression, anxiety, or psychotic symptoms. Participants will be recruited from clinics in the Metro Boston area. Aspects of TECC will be conducted in both Spanish and English to ensure wide access to care for multiple populations.

**Results:**

The results of the TECC study will be used to support or adapt this model of care and create training resources to ensure its dissemination. The study results will be posted on ClinicalTrials.gov, with primary outcomes related to changes in mood, anxiety, and stress, and secondary outcomes related to engagement, alliance, and satisfaction.

**Conclusions:**

TECC combines new digital mental health technology with updated clinical protocols and workflows designed to ensure patients can benefit from innovation in digital mental health. Supporting multiple languages, TECC is designed to ensure digital health equity and highlights how mobile health can bridge, not expand, gaps in care for underserved populations.

**International Registered Report Identifier (IRRID):**

PRR1-10.2196/23771

## Introduction

Both the short- and long-term effects of the COVID-19 pandemic are already leading to a paradigm shift across the health care industry [1-3]. The pandemic has underscored systemic health disparities, clinician shortages, and financial strain that highlight the need for new innovation [4-6]. As the field looks toward technology to offer accessible and cost-effective solutions, now is the time to consider how digitally informed care can provide improved outcomes while reducing access inequities for marginalized communities [7-10].

It is clear that the rapid adoption of technology in health care, especially telehealth, has been catalyzed by COVID-19. The mental health field has also adopted telehealth quickly with one clinic converting to 100% digital visits within 72 hours [11]. Data suggests the mental health field is now the largest user of telehealth [8], with prior barriers toward adoption now largely overcome [12-14]. Although concerns around telehealth visits remain, especially around the impact on the therapeutic alliance, research suggests a positive response from both patients and clinicians with no clear harm to alliance documented [15].

Yet, the increased uptake of telehealth can only address access to a limited extent, and the unmet need for mental health care continues to expand during the pandemic. Marginalized communities remain at a higher risk for exposure and with lower access to care. The Centers for Disease Control and Prevention recently reported that, regardless of age, Latinos are four times more likely to contract COVID-19 than their non-Latino White counterparts [16], and rates of psychosocial distress have increased most in the Latino community compared to any other group [17]. Although telehealth can increase access to care for some, meeting the rapidly rising needs of underserved populations—linguistic barriers, financial strains, culturally competent providers—will require more than just virtual face-to-face health care visits.

To increase access to care, the Technology Enabled Clinical Care (TECC) protocol will offer all aspects of treatment in Spanish and English. Research has shown that individuals with limited English proficiency are less likely to seek out specialty treatment like mental health care and, when diagnosed, have more severe long-term mental health complications [18]. With an estimated 25.4 million Americans who speak English less than “very well” [19], it is imperative that the mental health care field take a more active stance in tackling this unmet need. Although the app will be available in both Spanish and English languages, the Spanish version of the app will first be tested and reviewed for linguistic and cultural appropriateness before being used in clinical practice. This will be done by asking bilingual individuals for app feedback, with a focus on language and images used within the app

TECC proposes a new approach to care that uses the most therapeutic elements of face-to-face mental health treatment with the accessibility and innovation of smartphone technology toward the goal of offering effective care that is accessible to all communities, including minority and underserved populations. Evidence suggests that short-term therapies—often 8-12 weeks in length—are as effective as longer-term treatments in terms of both immediate and longitudinal outcomes [20]. However, these short-term therapies often fail to realize their potential because of clinician nonadherence to protocol and patient nonadherence to out-of-session exercises and homework. This nonadherence is not intentional by either party but rather a natural and well-documented pattern. Using technology, it is possible to help ensure treatment remains focused, with clear acknowledgment that overregulation and correction by technology would itself cause harm. This issue has already been raised by many mental health clinicians who report that some technology-based therapy programs limit the therapeutic approach and flexibility needed to help patients [21]. Thus, there is a need for technology to help guide care and ensure it remains on track while offering both patients and clinicians the autonomy to respond to unique care needs and clinical leads. The TECC protocol assessed in this study will offer such a hybrid approach and is informed by the research literature, focus groups with patients and clinicians, and our team’s experiences offering this form of care.

We realized the potential for this unique treatment while developing and implementing a *Digital Clinic*, an outpatient short-term mental health program based in Boston, Massachusetts. As outlined in previous publications, the Digital Clinic is a method of health care treatment that allows providers the ability to use digital technology to augment all aspects of care [22]. Initial successes of the digital clinic model are reflected in case reports, successful clinical outcomes, and positive patient feedback. However, the digital clinic framework offers a model for more complex care where the clinician uses digital tools like apps with less direct guidance and structure. This model is appropriate for many patients and especially those with treatment-resistant depression but requires more active investment from both clinician and patient. With this in mind, it may be possible to achieve the same outcomes for many patients who can benefit from brief and more structured evidence-based therapies while providing guidance for clinicians who seek more direction in using technology in care. Thus, although the theoretical model, hands-on experience, and actual framing of the Digital Clinic are imbued in this new protocol, the clinical focus of structured, educational, and guided care is what separates TECC from the original Digital Clinic model.

Taken from the Digital Clinic framework, this protocol will use a digital navigator (or technology specialist), a new team member who is able to offer technical support and assistance in ensuring the patient is able to thrive with technology use, so clinical visits remain focused on care needs. The digital navigators for this study will include research assistants who have completed the 10-hour digital navigator training. Briefly, the training consists of 5 modules: core smartphone skills, basic technology troubleshooting, app evaluation, clinical terminology and data interpretation, and engagement techniques. These modules will teach digital navigators how to perform their main responsibilities of selecting smartphone apps for care, troubleshooting technology issues for both client and clinician, and ensuring app data is understood by all parties [23].

Furthermore, TECC seeks to balance clinician and participant autonomy while providing some structure and treatment pathways. Thus, the protocol was specifically designed to empower clinicians and participants to use smartphone technology by customizing data collection of both mental health surveys as well as sensor data (if agreed upon) toward ensuring each participant’s unique experience is captured. Although each session of the protocol offers exercises related to aspects of the app, which the participant should complete between sessions, these activities are defined broadly to meet diverse clinical needs and offer clinicians therapeutic flexibility. For example, using the app toward goal setting and tracking will be applicable in almost any scenario.

The eight-session structure included in TECC will provide the flexibility for clients to learn about different treatment modalities to gain broad exposure to evidence-based therapeutic skills and techniques while still ensuring treatment is focused on personal needs. The clinical skills incorporated in TECC were chosen from current research done on short-term mental health treatments, studies designed specifically to understand marginalized communities, and app-based mental health interventions. The protocol consists of three main stages:

- Stage 1 “Creating the Clinical Foundation”: This stage will allow participants to learn and practice shared decision-making by cocreating treatment goals, identifying thought patterns, and identifying coping skills and defense mechanisms. The goal of this stage is for participants to develop a broad understanding of their thoughts and behaviors while beginning to identify strengths and need areas. Participants will also be introduced to clinical language and definitions for shared language to be established.
- Stage 2 “Building Clinical Skills”: Expanding from the skills learned in stage one, this stage will focus on teaching and practicing cognitive defusion and behavioral activation techniques. The goal for this stage is to expose participants to practical therapeutic skills that can be used across many situations and symptom management.
- Stage 3 “Solidifying Clinical Gains”: This stage introduces participants to mindfulness techniques, a diary card–like model, and patient activation and empowerment skills. The purpose of this stage is to ensure participants grasp all technical skills and clinical language reviewed throughout treatment to make informed autonomous decisions regarding treatment preferences.

This yearlong study will follow a prospective longitudinal cohort design where all participants will receive the same treatment and smartphone interventions. Based on our own Digital Clinic pilot data, we hypothesize participants will report a treatment response rate—defined as 50% symptom reduction—of improvements in anxiety and depression within the 8 weeks of treatment compared to their initial baseline. We also hypothesize that both rates of app engagement out of sessions, as well as therapeutic alliance measured during sessions, will be correlated with clinical improvements and suggest a mechanism of action for TECC. Furthermore the study will test the feasibility of providing technology-enabled mental health care within an outpatient clinical setting via virtual telehealth visits, explore the linguistic and cultural appropriateness of the smartphone app (mindLAMP) used in the study, and analyze the symptom changes of anxiety and depression for all study participants.

## Methods

### Study

The study will take place at Beth Israel Deaconess Medical Center (BIDMC), a Harvard affiliated teaching hospital in Boston, Massachusetts. The care team will include a psychiatrist, social worker, and digital navigator. The psychiatrist and social worker will provide clinical treatment, education, and support for all participants. The digital navigator, as previously discussed, will be available for technology support and mindLAMP training to other care team members and research participants.

For this study, 50 participants will be enrolled and will not be financially compensated for their participation in TECC. Given the success of the pilot Digital Clinic program, including 50 participants is feasible and necessary to collect a diverse sample and see significant effects of different techniques on clinical assessments of depression and anxiety. This study will be open to any adult (18 years or older) who is interested in using smartphone technology to augment their mental health care. Participants are required to meet the criteria for either mild to moderate depression or anxiety as measured on the Patient Health Questionnaire-9 (PHQ-9) or Generalized Anxiety Disorder-7 (GAD-7) scale. Participants must have daily access to a smartphone, be willing to download the mindLAMP app, use their smartphone for mental health care (both in and outside of session time), and be open to learning about an array of clinical modalities (dialectical behavior therapy [DBT], cognitive behavioral therapy [CBT], short psychodynamic therapies). For participants who do not own a smartphone, the care team will help individuals sign up for a free phone through the US Federal Government’s Project Lifeline if they are eligible. Participants must also be affiliated with a BIDMC or Beth Israel Lahey Health provider and be available for eight clinical sessions (telehealth) over the course of 2-4 months. Until the Spanish language version of the mindLAMP app has been properly vetted, participants must be able to read, write, and communicate in English at an eighth grade level. Recruitment efforts for TECC include digital flyers and brief informational sessions for referring providers.

### Interventions

Interventions for TECC will be employed through a multipronged approach including in-session care from clinicians, exposure and education to evidence-based therapies, use of the mindLAMP app, and technology support from the digital navigator.

At least 1 week before the initial clinical visit, participants will meet with the digital navigator to download the mindLAMP app, receive app training, and will be asked to begin completing the mood surveys (PHQ-9 and GAD-7) in mindLAMP on a daily basis. During the meeting, participants will be informed the app will not be used to offer emergency services between sessions and app responses will not be monitored outside of clinical sessions. A signed waiver will be presented to reflect this understanding.

**Figure 1:**
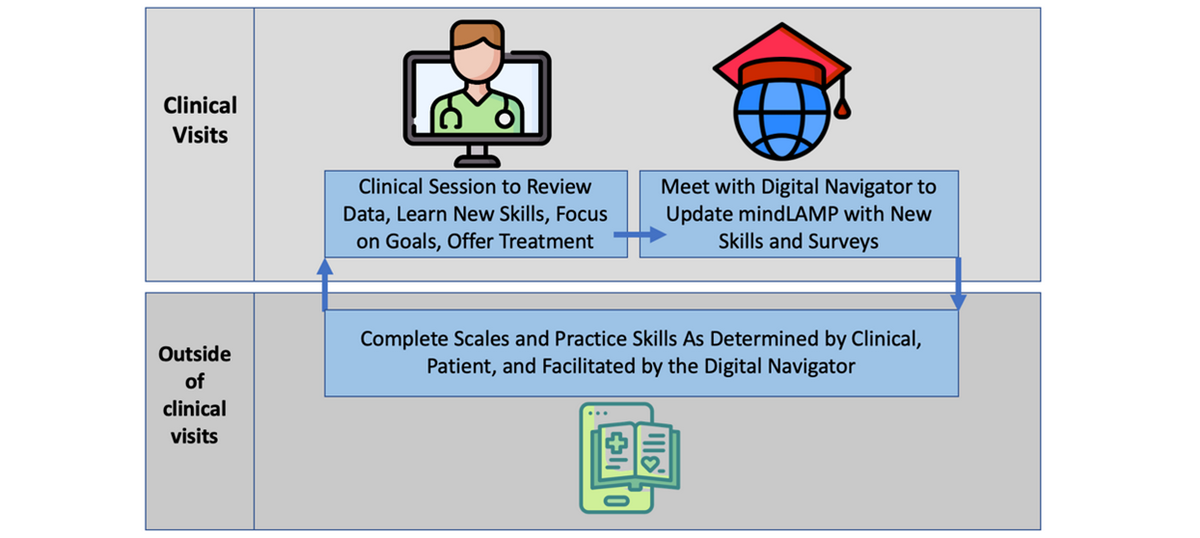
The Digital Clinic program structure including pre- and postclinical activities.

**Figure 2:**
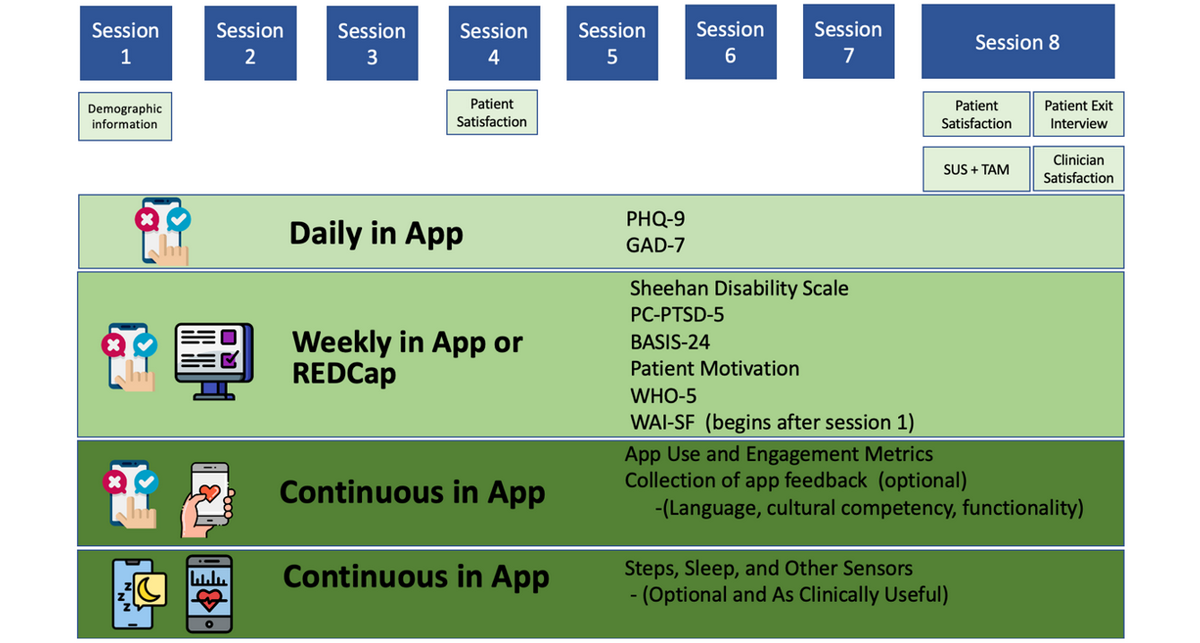
The Technology Enabled Clinical Care survey and data collection timeline. BASIS-24: Behavior and Symptom Identification Scale-24; GAD-7: Generalized Anxiety Disorder-7; PC-PTSD-5: Primary Care Posttraumatic Stress Disorder-5; PHQ-9: Patient Health Question-9; SUS: System Usability Scale; TAM: technology acceptance model; WAI-SF: Working Alliance Inventory–Short Form; WHO-5: World Health Organization Five.

The format for TECC sessions will follow the pre-established Digital Clinic structure (Figure 1). Specifically, before each clinical session, participants will be required to complete a variety of clinical surveys. These surveys will be made available through the clinic’s secured, research-based survey platform (REDCap, Vanderbilt University). The surveys (see Figure 2 for more details) are designed to help clinicians develop a comprehensive understanding of each participants progress throughout the study. Once the clinical portion of the session starts, any mindLAMP data collected will be reviewed and discussed. This step will ensure the data in mindLAMP accurately reflects the participant’s lived experience and ensure the personalized nature of each patient’s care is reflected in the session. Next, the clinician will discuss a predetermined clinical skill, and the clinician will work to relate the skill to the patient’s unique needs. The clinician will introduce the skill by describing its purpose, giving examples of when the skill can be used, and explain how the skill could impact the participants mental health needs. With clinician guidance, participants will then practice the skill, brainstorm scenarios in which the skill could be used, and review a skill-building activity (see Multimedia Appendix 1 for details). Activities will be conducted through skill-building modules (eg, mindfulness), informational worksheets, surveys, and free-writing opportunities that will be available through the app. Participants will be encouraged to complete the activities between sessions to strengthen their understanding and comfortability of use with each skill.

Upon completion of each clinical session, participants will meet with the digital navigator to address any technology-related questions or issues. The digital navigator will also work with participants to create any additional mood-tracking surveys and retrieve smartphone senor data (if applicable). The mindLAMP app will be used in and outside of the session to allow participants to track their daily moods (PHQ-9 and GAD-7 scales) and practice clinical skills (through activities in the app).

**Figure 3:**
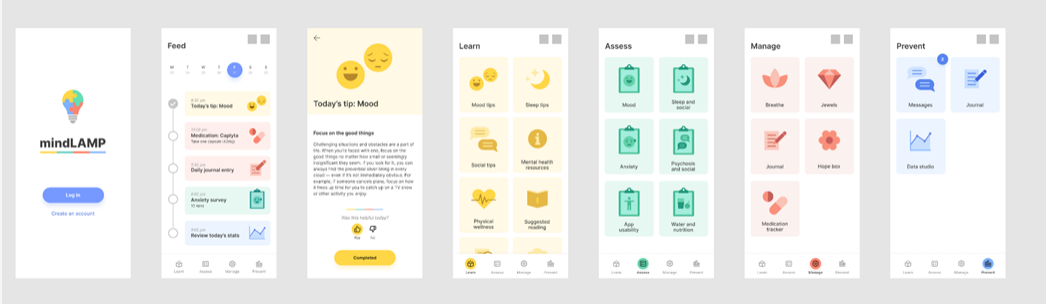
Screenshots of mindLAMP showing the Learn, Assess, Manage, and Prevent functions.

As previously mentioned, the mindLAMP app will be used to provide digitally augmented care [24]. Cocreated with patients and clinicians, mindLAMP is designed not to drive care decisions or protocolize treatment but, rather, to serve as a flexible tool to be deployed in a personalized manner to customize care for each person. To support this flexibility, mindLAMP allows users to create unique surveys specific to their needs and assign a schedule of times to be reminded [25]. Likewise, mindLAMP can capture diverse sensor data from the smartphone, ranging from step count, sleep, geolocation, and screen time, and the user can determine which, if any, are useful to capture. As an example, some patients may wish to track mood and sleep to determine if changing their sleep pattern may improve their mood [26]. Any use of such data will be determined by clinical needs and not the TECC protocol. mindLAMP also offers options for app interventions and supports a suite of resources that are customizable to the unique needs of users. The learning tab presents relevant tips and information, and the management tab has a series of tools and skill-building modules like mindfulness, defusion (from acceptance and commitment therapy [ACT]), goal setting, medication reminders, and emergency planning. Not all learning and management offerings will be relevant, or even useful, for all patients, and the goal of care is to help match the right elements of mindLAMP at the right time for each patient—much like in traditional medication management or therapy care. As a tool, mindLAMP aims to be accessible on Apple and Android smartphones as well as on an internet browser (ie, computers) and can support multiple languages including Spanish and English. In this protocol, mindLAMP is used in a structured manner, with each week using different aspects of the app, but personalization and customization are still part of each week’s use, as outlined in later sections. Figure 3 provides several examples of the mindLAMP interface. A set of handouts is also included in Multimedia Appendix 1 that can be used as nondigital *backups*, in case a patient has a technical issue, and can be mailed or emailed to each patient in TECC.

At the end of the study, participants will be provided a paper or electronic packet of all therapeutic skills learned, activities practiced, and mindLAMP data collected. Participants will also be allowed to keep using the app as a self-help tool or delete the app completely. A copy of clinical metrics and results will be offered to participants’ referring provider to ensure transparency and continuation of care. In general, TECC clinical sessions and digital navigator meetings can be conducted in person or through a video platform. However, due to COVID-19, all TECC-related participant meetings for the next year will be held over a Health Insurance Portability and Accountability Act–compliant video platform to maintain social distancing practices.

**Figure 4:**
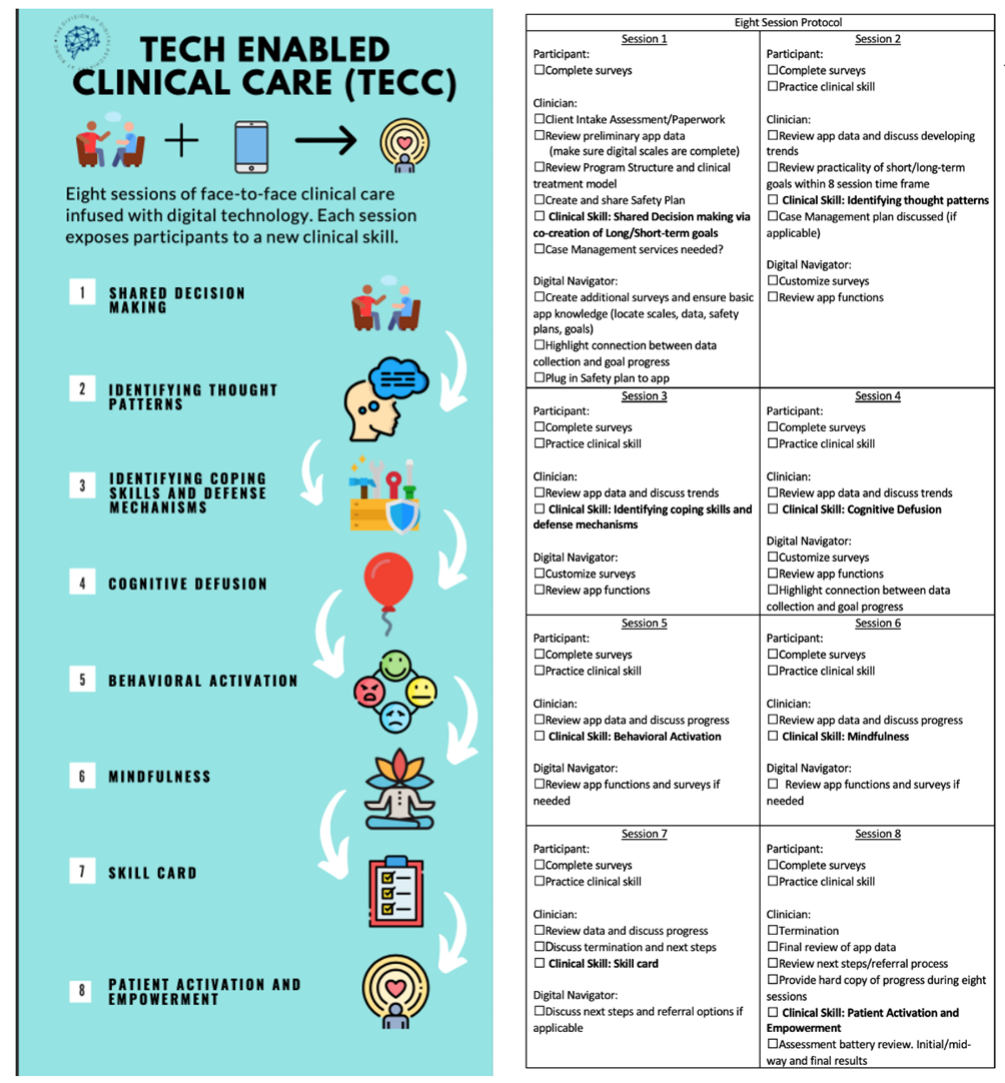
Overview of clinical sessions (left) and session-specific checklist (right).

As previously mentioned, TECC follows an eight-session template (Figure 4), which provides a step-by-step guide for participants, clinicians, and digital navigators.

### Session One: Shared Decision Making

**Figure 5:**
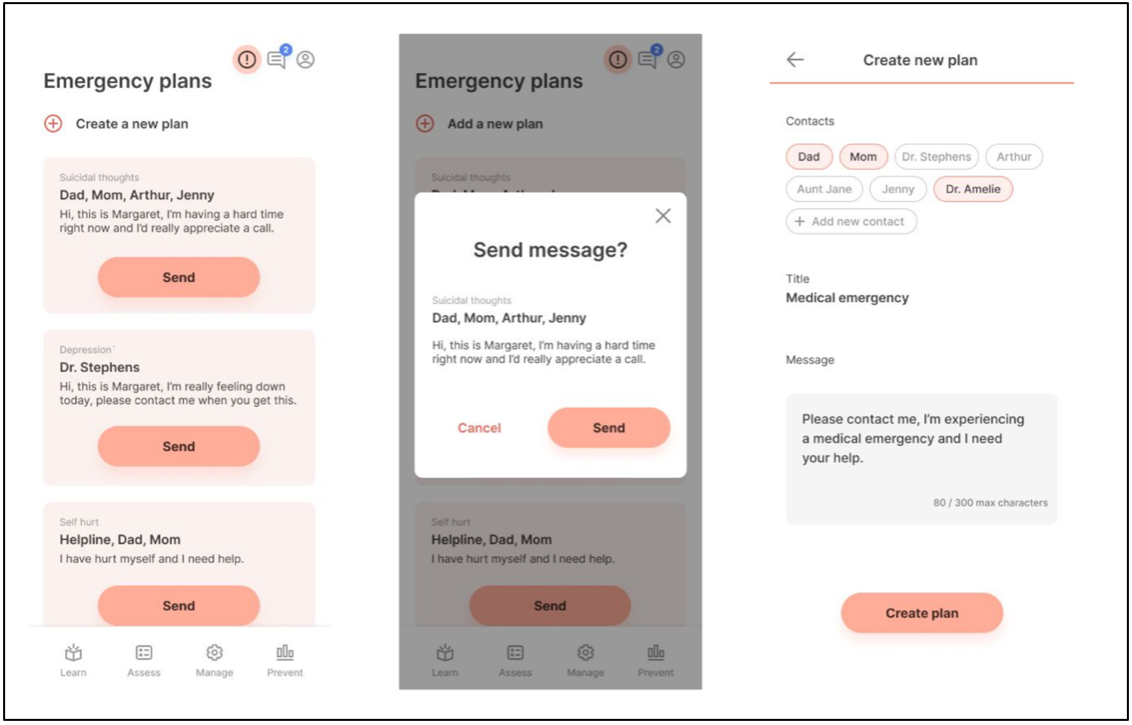
Example of one aspect of the safety planning activity as seen in mindLAMP.

As with most initial therapeutic sessions, a client assessment will be completed, a safety plan created, and feasibility of short- and long-term goals will be addressed. (Please note, the safety plan used in TECC was not created by members of the research team and was borrowed from our affiliated hospital’s psychiatric department.) Figure 5 shows an example of how the safety plan can appear in mindLAMP. After safety planning, the study time frame, expectations, and structure will be reviewed with participants. Since the study will be providing clinical treatment in a time-limited manner, the need for supplemental case management services will also be evaluated, and education and resources may be provided; however, connections to services cannot be guaranteed. For the remainder of the session, the skill of shared decision making (SDM) will be discussed.

**Figure 6:**
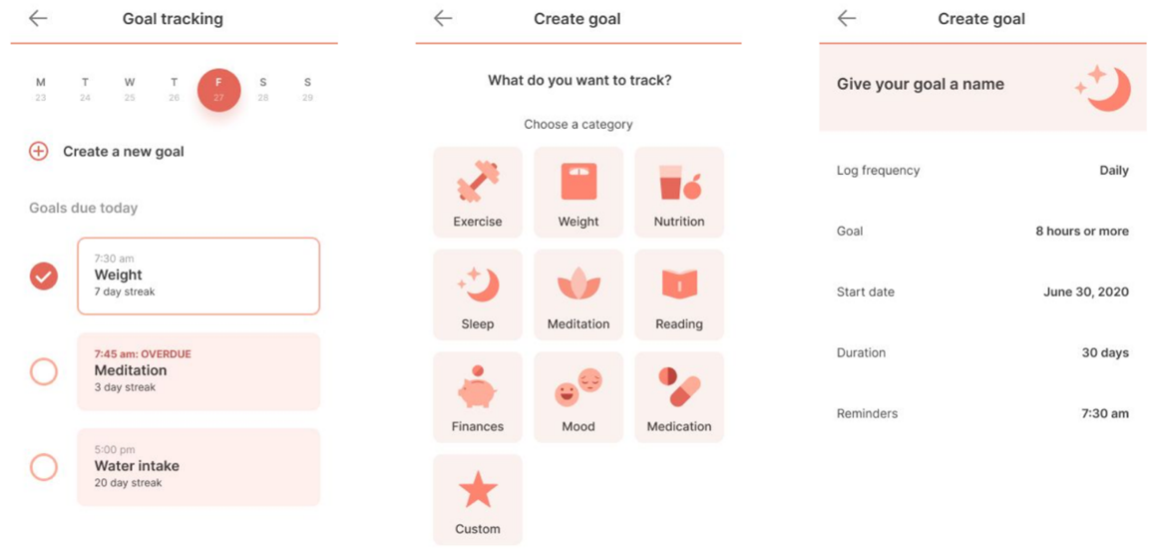
Goal tracking in mindLAMP.

For the purposes of this study, SDM will be defined as the process when provider and client review available treatment options and when clients are encouraged to select the treatment plan that best fits their personal preferences [27]. Previous studies combining app technology and SDM interventions have been mixed [28], and several limitations have been identified, such as time constraints and provider bias toward which patient could benefit and which situation calls for the intervention [29]. However, our team will use this tool for the purposes of discussing and creating short-term therapeutic goals with participants. In addition to clinical conversations, SDM practices will be reinforced through out-of-session activities available through the app. Figure 6 provides an example of one goal-tracking activity as it appears in mindLAMP.

Once each of the clinical visits are over, participants will meet with the digital navigator to review any app-related questions or concerns. The digital navigator will also work with participants and clinicians to ensure that a connection between app data and clinical goals is clear.

### Session Two: Identifying Thought Patterns

The second skill will focus specifically on understanding and identifying thought patterns. CBT will be introduced using a core CBT principle, ensuring information is presented in a structured and educational method, which allows for in-the-moment focus and client participation [30]. Again, mindLAMP will assist in this process by providing clients with out-of-session education and activities. mindLAMP activities will include documenting, describing, and labeling different types of thinking patterns.

**Figure 7:**
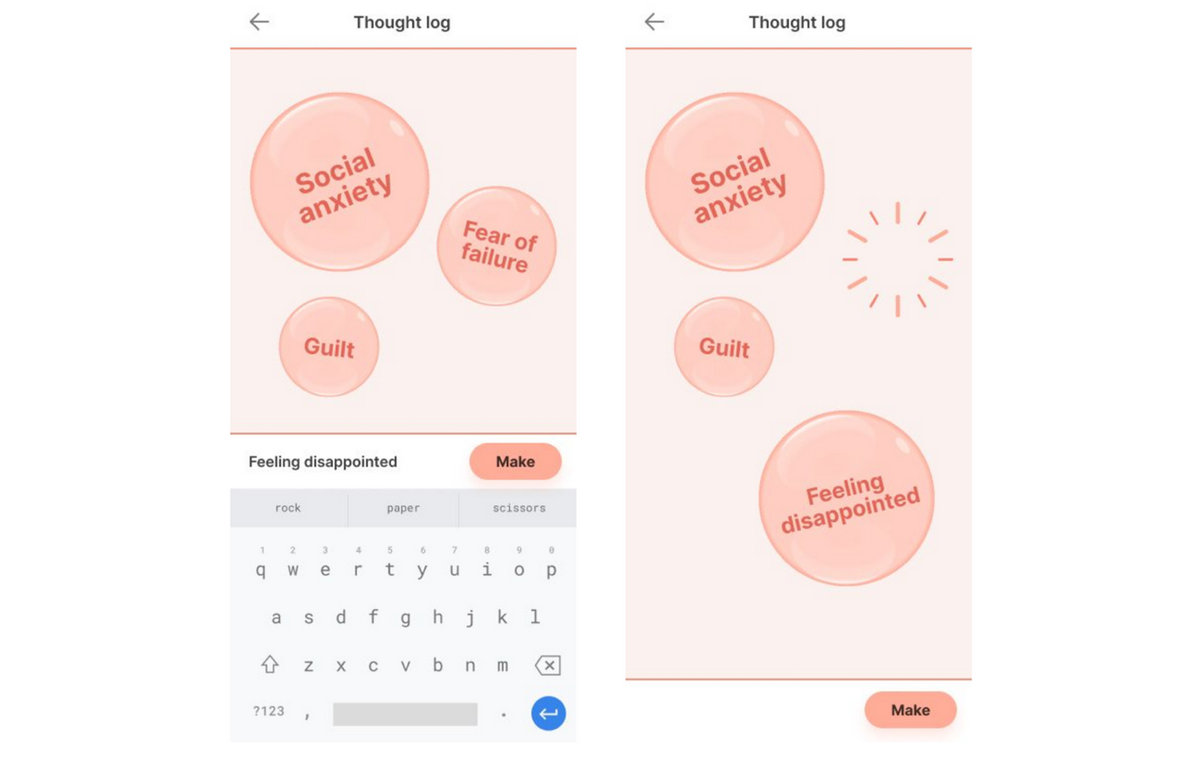
The thought log allows participants to label and track thought patterns in real time.

Studies have shown that CBT smartphone apps have therapeutic potential [31]; however, like many apps, high user engagement and sufficient long-term outcomes seem to be the top barriers for effective CBT app outcomes [32]. User engagement will be closely monitored through review of out-of-session activities, incorporating participant feedback when applicable, and monitoring participants overall engagement to care. Figure 7 provides an example of a mindLAMP activity that can be used for this skill.

### Session Three: Identify Coping Skills and Defense Mechanisms 

In session one, a safety plan was cocreated using participant’s language and clinician guidance. In this session, beneficial coping skills will now be identified and discussed in more depth, allowing coping skills to be the main clinical focal point.

To the best of our team’s knowledge, there are no psychodynamic mental health apps that have been widely tested and made available to the public. Providing short-term psychodynamic treatment presents many challenges (time constraints, adequate provider training, bias toward newer clinical modalities); however, recent studies continue to test new ways psychodynamic practices can be beneficial [33]. Therefore, this session will help participants learn and identify some defense mechanisms and help bridge the connection between defense mechanisms and coping skills. The mindLAMP activity will include a list of defense mechanisms (terms, definitions, and examples) and ask participants to link their preferred coping skill with a defense mechanism (if applicable).

### Session Four: Cognitive Defusion

In this session, participants will learn therapeutic skills that offer broad applicability. Specifically, they will learn cognitive defusion, an ACT intervention, which asks participants to notice their thoughts (nonjudgmentally and without identifying with them), acknowledge them, and then let those thoughts leave one’s mind [34].

**Figure 8:**
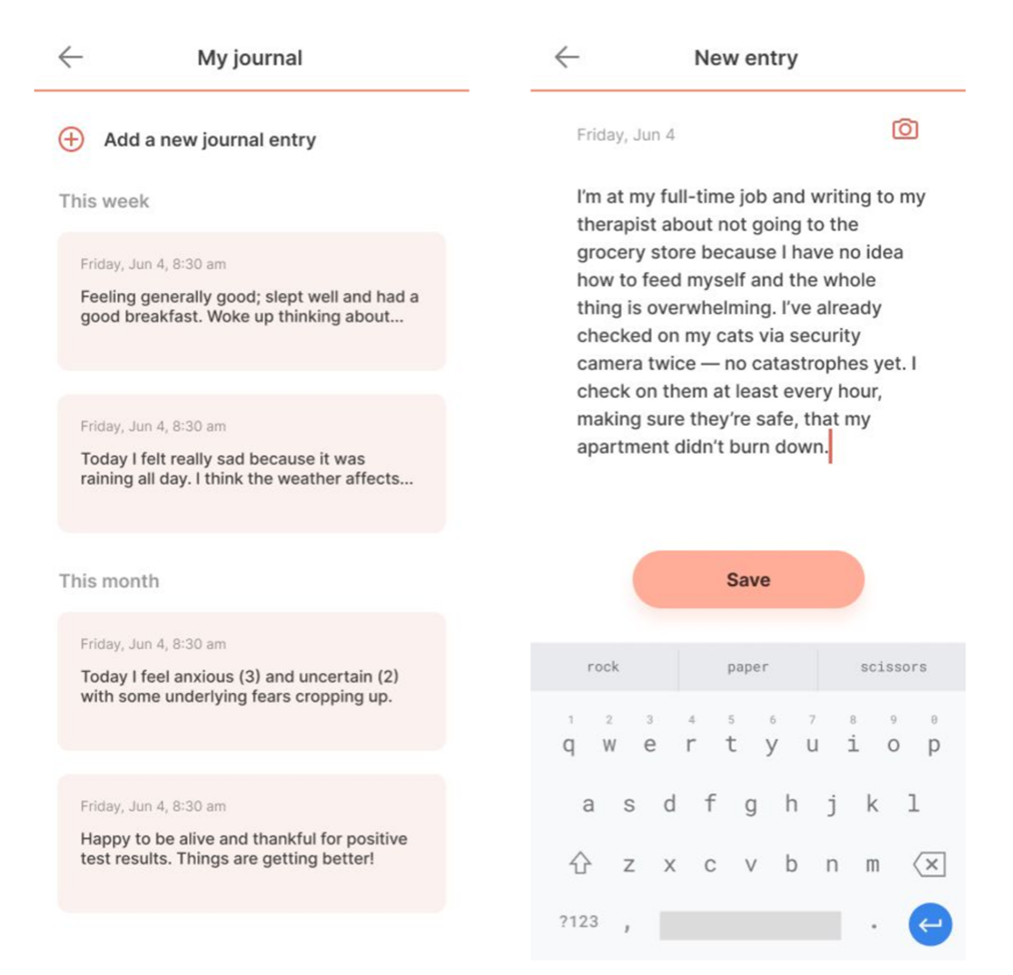
Example of mindLAMP’s journal section, which allows participants space to freely reflect on experiences.

ACT techniques can be taught in a time-limited fashion, and the straightforward approach can be practiced independently once understood. In a 2-week trial, Levin et al [35] found that participants who used an app intervention to practice ACT skills believed the method was helpful and accessible; furthermore, participants’ depression, anxiety, and psychological flexibility also showed significant improvement. Similar to other clinical skills, cognitive defusion will be practiced through the mindLAMP app by listing the steps of cognitive defusion, asking participants to practice the skill, and then providing space for participants to summarize their experience (see Figure 8 for example). Aspects of session four and the mindLAMP thought log (Figure 7) can be used as well.

### Session Five: Behavioral Activation

Session five will continue to develop participants’ tool kit of therapeutic skills. Modern day behavioral activation techniques include encouraging clients to become aware of avoidance behaviors and places extra emphasis on understanding environmental factors rather than thought patterns [36]. The broad applicability of these skills makes them ideal for TECC and meeting the needs for diverse populations.

Although research on behavioral activation through mobile apps is nascent, one qualitative study found that, when using app interventions, participants felt their treatment was more accessible, were more motivated to engage, and felt security [37]. The behavioral activation mindLAMP activity will include a list of step-by-step instructions and provide space for participants to share their experience.

### Session Six: Mindfulness

**Figure 9:**
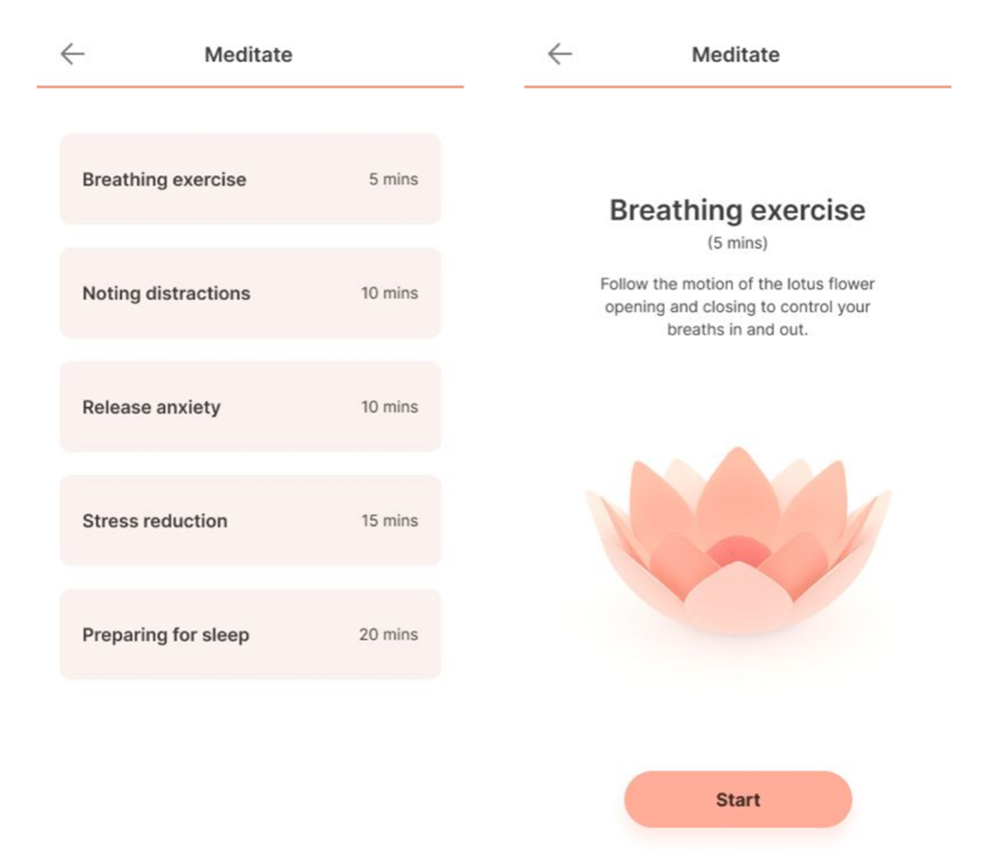
Example of one of the guided mindfulness exercises offered in mindLAMP.

Broadly, mindfulness skills ask participants to focus on aspects of their present state as well as factors in their immediate surroundings in a nonjudgmental way. Additionally, practicing mindfulness has been thought to enhance self-awareness, openness to new experiences, and provide users with a deeper understanding of self [38]. Mobile technology studies, including a 2018 randomized trial and a separate efficacy-based investigation, found mobile app interventions could reduce stress and irritability, and yielded statistically significant efficacy measures [39,40]. Mindfulness techniques will be practiced through mindLAMP by providing instructions on how to engage with the technique; participants will again have the option to document their experience (see Figure 9 for example).

### Session Seven: Skills Card

This session introduces participants to a means to reflect on their mental health and build emotional self-awareness. Similar to a DBT diary card (where individuals document and monitor symptoms and behavioral skills used) participants will cocreate a skills card to help practice, identify, and monitor interventions learned and used in their daily lives. App-related DBT studies have shown potential in allowing users to learn and practice a wide range of skills; however, major barriers include app navigation challenges and difficulties understanding interventions without prior DBT exposure or clinical support [41,42]. Nonetheless, increasing symptom awareness and tracking, via diary cards, have been shown to be helpful for various conditions, including those in jeopardy of heart failure, chronic bronchitis, and pulmonary diseases [43,44].

### Session Eight: Patient Activation and Empowering

Based on the Right Question Project, this skill will empower participants to take an engaged role in care by actively asking questions that will encourage clients to participate in the decision-making process [45]. Additionally, the clinical team will pay special attention to ensure culturally appropriate language and values are considered when practicing this skill. Research on apps that support this particular skill is limited. mindLAMP will provide support by encouraging participants to practice advocating for themselves in clinical care by using the skills they have learned throughout treatment and using them outside of clinical sessions.

### End of Study

At the end of the eight-session study, handouts of the data collected and skills learned throughout the study will be given to participants and briefly reviewed a final time. Participants will also be asked to complete a more extensive patient satisfaction survey, which will assess both the clinical sessions and general review of the mindLAMP app. Finally, before ending the study, participants will have a chance to provide verbal feedback to all care team members if they choose. Results and graphs created by participants in mindLAMP will also be made available to participant’s referring provider to assist with continuation of care and build a connection between mental health and additional health care treatment.

### Study Measures

Since TECC was designed to collect a number of clinical surveys throughout treatment, the timeline for survey collection was carefully crafted to decrease oversaturation (see Figure 2 for survey timeline). Specifically, participants will be asked to complete the PHQ-9 (which measures depression rates from minimal depression to severe depression) and the GAD-7 scale (a widely accepted anxiety scale that evaluates scores as either mild, moderate, or severe anxiety) on a daily basis [46,47]. Both the PHQ-9 and GAD-7 scales will be loaded on mindLAMP and become available to participants upon downloading.

Prior to each clinical visit, participants will briefly meet with a research assistant who will provide participants with a secure link (via REDCap) to weekly clinical surveys. This could be done via mindLAMP, but we will use a different platform for the purposes of this study. Those presession surveys include the Sheehan Disability Scale, which measures the effects of poor mental health across three domains: work and school, family, and social life [48]; the Primary Care Posttraumatic Stress Disorder-5, which assess for posttraumatic stress disorder [49]; the Behavior and Symptom Identification Scale-24, a symptom assessment scale [50]; a patient motivation scale, to asses participants commitment to change through treatment; the World Health Organization Five, a well-being assessment scale [51]; and the Working Alliance Inventory–Short Form, a scale to assess the alliance a patient feels toward the clinician that is known to be a predictor of treatment success (this scale will begin after session one) [52].

Additional nonweekly scales include a participant satisfaction scale, which will be given after session four and at the end of treatment, and the System Usability Scale, which is offered during the termination process and is a well-accepted tool to assess satisfaction around use of mindLAMP and TECC [53]. The technology acceptance model, a well-known implementation science framework, will also be used to assess feasibility of technology-infused care [54]. Since the mindLAMP app is able to automatically capture data about when and how it is used, the study will offer a unique data set on longitudinal use patterns and engagement. A provider-specific satisfaction survey will be given to all referring providers at the end of the study to improve the referral process, assess the relevance of clinical data shared, and streamline continuation of care. Other metrics in this study include engagement with the app, as measured by number of log-ins each day, as well as time spent on each element of the app during each log-in. Basic demographics and measures of socioeconomic status will also be collected during the intake process.

### Further Study Details

All participants will complete a written informed consent for this voluntary study. Participants will be able to withdraw from TECC anytime, just as they can withdraw from routine care at any time. Participants who miss four consecutive sessions will be asked to restart the protocol. During the study, if a participant’s clinical presentation significantly worsens, the clinical team will re-evaluate the participant’s involvement in the study. Intent-to-treat analysis will be applied so that data from all participants, even those who drop out, will be included in the outcomes.

The mindLAMP app meets federal privacy and security requirements, and has been institutional review board (IRB)–approved for use in clinical research. All data collected by mindLAMP will be securely transmitted directly to a server within the hospital network. Additionally, after receiving IRB approval, the TECC protocol will be registered with ClinicalTrials.gov.

## Results

**Figure 10:**
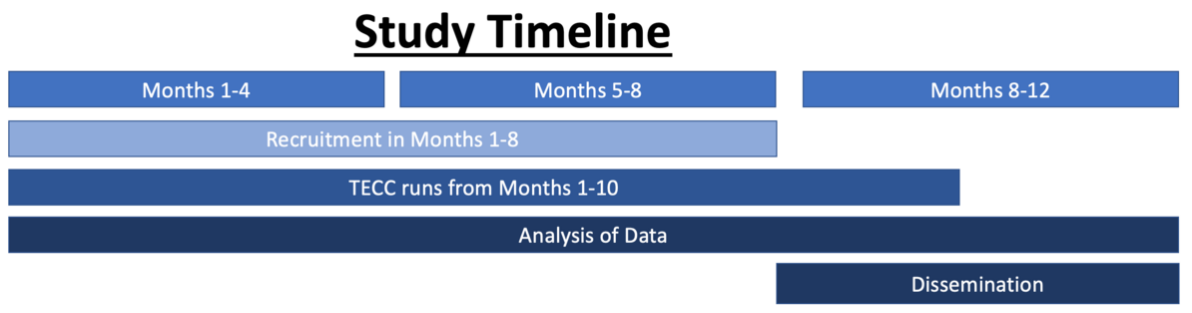
Study timeline. TECC: Technology Enabled Clinical Care.

The results of the TECC study will be used to support or adapt this model of care and create training resources to ensure its dissemination. The 1-year study timeline for TECC includes continuous recruitment and enrollment efforts until the sample size is reached; 10 months of clinical sessions, continuous collection of data, and in-session review of clinical metrics; several months spent analyzing data; and dissemination of study results (see Figure 10 for details).

### Analysis

Analysis will include calculation of the effect size of TECC to reduce symptoms related to stress, anxiety, and depression. Analysis will also focus on assessing association between baseline factors, race, language, and education, as well as dynamic factors like working alliance and technology engagement patterns with changes in symptoms and use and adherence to the TECC protocol. We will assess relationships between these factors and clinical changes using generalized linear models and multiple regression analyses. Tests for dropout effects will be performed to see if outcome and covariate measures are associated with differences in engagement. If there are significant dropout effects, a logistic dropout probability model will be fit using the significant dropout outcomes and covariates measured at the start of the stage, and inverse probability weighting will be used with these model probabilities to account for dropout in each of the models. The age-matched control group will be used to compare changes related to clinical scores and calculate effect sizes using means and SDs.

## Discussion

The eight-session TECC protocol outlined in this paper provides one solution for the ever-growing need for high-quality, evidence-based, time-limited mental health care. Specifically, the TECC protocol will use smartphone technology as a tool to enhance face-to-face (virtual) clinical sessions for clinicians and participants. TECC is designed to allow clinicians and participants a way to monitor customized information from surveys to sensors and bring this data into clinical visits toward improving outcomes. The mindLAMP app used in TECC also offers a flexible suite of app-based interventions that can be used to augment care outside of sessions. Given the digital nature of TECC, it is easy to share a comprehensive digital record of participant progress across the study and ensure transitions in care can be as seamless as possible. Results from this study will thus offer important data in understanding how to best share and implement TECC toward further improving access to care.

The bedrock of the TECC treatment protocol aims to improve clinical care by exposing participants to an array of different therapeutic modalities. With increased exposure to foundational clinical principles and basic understanding of how to use clinical skills, individuals will learn to identify the clinical modalities and interventions that are most effective for them. By the end of treatment, participants will have developed the skills needed to become more actively engaged in their mental health care and be better equipped to advocate for their mental health needs in the future. Even when the TECC study ends, patients can continue to use mindLAMP as a self-help tool and build off the skills and advances they made while in care.

By using the mindLAMP app, which will reside on a participant’s personal mobile device, individuals will be continuously connected and engaged with treatment, something less feasible with nontechnology augmented care. Encouraging participants to frequently engage with treatment in an inconspicuous way (via their smartphone) may decrease mental health stigma, as treatment will be seamlessly woven into already established behaviors (using smartphones in public). Additionally, by increasing app engagement, clinicians will be provided an array of individualized clinical measures and important participant narratives regrading treatment progress. These narratives will be reviewed with participants during clinical sessions and help clinicians customize activities for outside of the session by using individuals’ preferred terminology and preferences. The customization and flexibility built into the model will help ensure high app engagement, as past research has shown high app engagement can correlate with higher clinical outcomes [55]. Furthermore, by allowing flexibility and customization of language used during skill-building activities, the study aims to limit dropout rates, as patients tend to drop out of care less when receiving treatment that matches their personal preferences [56].

TECC also offers unique potential for underserved populations. At the time of this writing, there were an estimated 10,000 mental health apps available to consumers [57]. Using digital tools, specifically smartphone apps, is a highly scalable solution to help ensure language-appropriate resources are available to all communities. Despite this promise, access to diverse language technology, especially in apps, continues to be limited. A 2018 review of diabetes-related Spanish language apps found only 25% of Android and 19% of IOS apps allowed users to fully interact with the app in Spanish [58]. Language barriers are not the only issue that contributes to health inequities, and much has been written about these factors that are collectively known as social determinants of health [59-62]; however, before fully incorporating a multi-language app into care, the team will ensure the Spanish Beta version of the app is culturally and linguistically appropriate for Spanish-speaking individuals. This will be done through feedback gathered from community members, participants, and providers who are well versed in the Spanish language and culture. Once the additional language version of the app is available in early 2021, the TECC protocol will be conducted again in Spanish.

It is undeniable that recent circumstances have forced providers and patients to use digital technologies for continued care, personalized treatment, and remote health care appointments to a degree that would not have been considered feasible in the pre–COVID-19 era. Since technology devices and digital platforms will continuously evolve over time, now is the moment to test this hybrid care model to offer innovative treatments that are effective and available to and for as many people as possible.

## Appendix

Multimedia Appendix 1 Handouts.

## Abbreviations

ACT: acceptance and commitment therapy
BIDMC: Beth Israel Deaconess Medical Center
CBT: cognitive behavioral therapy
DBT: dialectical behavior therapy
GAD-7: Generalized Anxiety Disorder-7
IRB: institutional review board
PHQ-9: Patient Health Questionnaire-9
SDM: shared decision making
SPIRIT: Standard Protocol Items: Recommendations for Interventional Trials
TECC: Technology Enabled Clinical Care

## References

[1] Adams JG, Walls RM (2020). Supporting the health care workforce during the COVID-19 global epidemic. JAMA.

[2] Gates B (2020). Responding to Covid-19 - a once-in-a-century pandemic?. N Engl J Med.

[3] Rajkumar RP (2020). COVID-19 and mental health: a review of the existing literature. Asian J Psychiatr.

[4] Khullar D, Bond AM, Schpero WL (2020). COVID-19 and the financial health of US hospitals. JAMA.

[5] Selden TM, Berdahl TA (2020). COVID-19 and racial/ethnic disparities in health risk, employment, and household composition. Health Aff (Millwood).

[6] Zhang X, Lin D, Pforsich H, Lin VW (2020). Physician workforce in the United States of America: forecasting nationwide shortages. Hum Resour Health.

[7] Moazzami B, Razavi-Khorasani N, Dooghaie Moghadam A, Farokhi E, Rezaei N (2020). COVID-19 and telemedicine: immediate action required for maintaining healthcare providers well-being. J Clin Virol.

[8] Torous J, Jän Myrick K, Rauseo-Ricupero N, Firth J (2020). Digital mental health and COVID-19: using technology today to accelerate the curve on access and quality tomorrow. JMIR Ment Health.

[9] Webb Hooper M, Nápoles AM, Pérez-Stable EJ (2020). COVID-19 and racial/ethnic disparities. JAMA.

[10] Boserup B, McKenney M, Elkbuli A (2020). The financial strain placed on America's hospitals in the wake of the COVID-19 pandemic. Am J Emerg Med.

[11] Yellowlees P, Nakagawa K, Pakyurek M, Hanson A, Elder J, Kales HC (2020). Rapid conversion of an outpatient psychiatric clinic to a 100% virtual telepsychiatry clinic in response to COVID-19. Psychiatr Serv.

[12] Jacob C, Sanchez-Vazquez A, Ivory C (2020). Social, organizational, and technological factors impacting clinicians' adoption of mobile health tools: systematic literature review. JMIR mHealth uHealth.

[13] Konttila J, Siira H, Kyngäs H, Lahtinen M, Elo S, Kääriäinen M, Kaakinen P, Oikarinen A, Yamakawa M, Fukui S, Utsumi M, Higami Y, Higuchi A, Mikkonen K (2019). Healthcare professionals' competence in digitalisation: A systematic review. J Clin Nurs.

[14] Shank N, Willborn E, Pytlikzillig K, Noel H (2012). Electronic health records: eliciting behavioral health providers' beliefs. Community Ment Health J.

[15] Torous J, Wykes T (2020). Opportunities from the coronavirus disease 2019 pandemic for transforming psychiatric care with telehealth. JAMA Psychiatry.

[16] (2020). Health equity considerations and racial and ethnic minority groups. Centers for Disease Control and Prevention.

[17] McGinty EE, Presskreischer R, Han H, Barry CL (2020). Psychological distress and loneliness reported by US adults in 2018 and April 2020. JAMA.

[18] Bauer AM, Chen C, Alegría M (2010). English language proficiency and mental health service use among Latino and Asian Americans with mental disorders. Med Care.

[19] (2015). Detailed languages spoken at home and ability to speak English for the population 5 years and over: 2009-2013. United States Census Bureau.

[20] Goodyer IM, Reynolds S, Barrett B, Byford S, Dubicka B, Hill J, Holland F, Kelvin R, Midgley N, Roberts C, Senior R, Target M, Widmer B, Wilkinson P, Fonagy P (2017). Cognitive behavioural therapy and short-term psychoanalytical psychotherapy versus a brief psychosocial intervention in adolescents with unipolar major depressive disorder (IMPACT): a multicentre, pragmatic, observer-blind, randomised controlled superiority trial. Lancet Psychiatry.

[21] Lattie E, Nicholas J, Knapp AA, Skerl JJ, Kaiser SM, Mohr DC (2020). Opportunities for and tensions surrounding the use of technology-enabled mental health services in community mental health care. Adm Policy Ment Health.

[22] Rodriguez-Villa E, Rauseo-Ricupero N, Camacho E, Wisniewski H, Keshavan M, Torous J (2020). The digital clinic: implementing technology and augmenting care for mental health. Gen Hosp Psychiatry.

[23] Wisniewski H, Gorrindo  T, Rauseo-Ricupero N, Hilty D, Torous J (2020). The role of digital navigators in promoting clinical care and technology integration into practice. Digital Biomarkers.

[24] Torous J, Wisniewski H, Bird B, Carpenter E, David G, Elejalde E, Fulford D, Guimond S, Hays R, Henson P, Hoffman L, Lim C, Menon M, Noel V, Pearson J, Peterson R, Susheela A, Troy H, Vaidyam A, Weizenbaum E, Naslund JA, Keshavan M (2019). Creating a digital health smartphone app and digital phenotyping platform for mental health and diverse healthcare needs: an interdisciplinary and collaborative approach. J Technol Behav Sci.

[25] Vaidyam A, Roux S, Torous J (2020). Patient innovation in investigating the effects of environmental pollution in schizophrenia: case report of digital phenotyping beyond apps. JMIR Ment Health.

[26] Wisniewski H, Henson P, Torous J (2019). Using a smartphone app to identify clinically relevant behavior trends symptom report, cognition scores, and exercise levels: a case series. Front Psychiatry.

[27] Elwyn G, Laitner S, Coulter A, Walker E, Watson P, Thomson R (2010). Implementing shared decision making in the NHS. BMJ.

[28] Korsbek L, Tønder ES (2016). Momentum: a smartphone application to support shared decision making for people using mental health services. Psychiatr Rehabil J.

[29] Légaré F, Ratté S, Gravel K, Graham IS (2008). Barriers and facilitators to implementing shared decision-making in clinical practice: update of a systematic review of health professionals' perceptions. Patient Educ Couns.

[30] Beck JS (2011). Cognitive Behavior Therapy, Second Edition: Basics and Beyond.

[31] Rathbone AL, Clarry L, Prescott J (2017). Assessing the efficacy of mobile health apps using the basic principles of cognitive behavioral therapy: systematic review. J Med Internet Res.

[32] Kinderman P, Hagan P, King S, Bowman J, Chahal J, Gan L, McKnight R, Waldon C, Smith M, Gilbertson J, Tai S (2016). The feasibility and effectiveness of Catch It, an innovative CBT smartphone app. BJPsych Open.

[33] Chen CK, Ingenito CP, Kehn MM, Nehrig N, Abraham KS (2019). Implementing brief dynamic interpersonal therapy (DIT) in a VA medical center. J Ment Health.

[34] Blackledge J (2017). Disrupting verbal processes: cognitive defusion in acceptance and commitment therapy and other mindfulness-based psychotherapies. Psychological Rec.

[35] Levin ME, Haeger J, Pierce B, Cruz RA (2017). Evaluating an adjunctive mobile app to enhance psychological flexibility in acceptance and commitment therapy. Behav Modif.

[36] Jacobson NS, Martell CR, Dimidjian S (2001). Behavioral activation treatment for depression: returning to contextual roots. Clin Psychol Sci Pract.

[37] Ly K, Janni E, Wrede R, Sedem M, Donker T, Carlbring P, Andersson G (2015). Experiences of a guided smartphone-based behavioral activation therapy for depression: a qualitative study. Internet Interventions.

[38] Langer E (1992). Matters of mind: mindfulness/mindlessness in perspective. Consciousness Cogn.

[39] Economides M, Martman J, Bell MJ, Sanderson B (2018). Improvements in stress, affect, and irritability following brief use of a mindfulness-based smartphone app: a randomized controlled trial. Mindfulness (N Y).

[40] van Emmerik AAP, Berings F, Lancee J (2018). Efficacy of a mindfulness-based mobile application: a randomized waiting-list controlled trial. Mindfulness (N Y).

[41] Washburn M, Parrish DE (2013). DBT self-help application for mobile devices. J Technol Hum Services.

[42] Suñol J, María Panisello J, Castell E, Juan Tárraga López P, Sánchez C, Pérez V (2017). "Medtep DBT": a dialectical behavior therapy native app and web platform for borderline personality disorder patients and their therapists. Universal J Public Health.

[43] Park L, Dracup K, Whooley MA, McCulloch C, Jin C, Moser DK, Clark RA, Pelter MM, Biddle M, Howie Esquivel J (2017). Symptom diary use and improved survival for patients with heart failure. Circ Heart Fail.

[44] Llor C, Moragas A, Miravitlles M, ESAB study (2012). Usefulness of a patient symptom diary card in the monitoring of exacerbations of chronic bronchitis and chronic obstructive pulmonary disease. Int J Clin Pract.

[45] Staples LH (1990). Powerful ideas about empowerment. Adm Soc Work.

[46] Kroenke K, Spitzer R L, Williams J B (2001). The PHQ-9: validity of a brief depression severity measure. J Gen Intern Med.

[47] Spitzer RL, Kroenke K, Williams JBW, Löwe B (2006). A brief measure for assessing generalized anxiety disorder: the GAD-7. Arch Intern Med.

[48] Rush JA (2000). Handbook of Psychiatric Measures.

[49] Prins A, Bovin MJ, Kimerling R, Kaloupek DG, Marx BP, Kaiser AP, Schnurr PP (2015). Primary Care PTSD Screen for DSM-5 (PC-PTSD-5). US Department of Veteran Affairs.

[50] Eisen SV, Normand S, Belanger AJ, Spiro A, Esch D (2004). The Revised Behavior and Symptom Identification Scale (BASIS-R): reliability and validity. Med Care.

[51] Topp CW, Østergaard SD, Søndergaard S, Bech P (2015). The WHO-5 Well-Being Index: a systematic review of the literature. Psychother Psychosom.

[52] Munder T, Wilmers F, Leonhart R, Linster HW, Barth J (2010). Working Alliance Inventory-Short Revised (WAI-SR): psychometric properties in outpatients and inpatients. Clin Psychol Psychother.

[53] Finstad K (2006). The system usability scale and non-native English speakers. J Usability Stud.

[54] Szajna B (1996). Empirical evaluation of the revised technology acceptance model. Manage Sci.

[55] Mattila E, Lappalainen R, Välkkynen P, Sairanen E, Lappalainen P, Karhunen L, Peuhkuri K, Korpela R, Kolehmainen M, Ermes M (2016). Usage and dose response of a mobile acceptance and commitment therapy app: secondary analysis of the intervention arm of a randomized controlled trial. JMIR mHealth uHealth.

[56] Windle E, Tee H, Sabitova A, Jovanovic N, Priebe S, Carr C (2020). Association of patient treatment preference with dropout and clinical outcomes in adult psychosocial mental health interventions: a systematic review and meta-analysis. JAMA Psychiatry.

[57] Torous J, Roberts LW (2017). Needed innovation in digital health and smartphone applications for mental health: transparency and trust. JAMA Psychiatry.

[58] Rodriguez J, Singh K (2018). The Spanish availability and readability of diabetes apps. J Diabetes Sci Technol.

[59] Bishop-Fitzpatrick L, Kind AJH (2017). A scoping review of health disparities in Autism Spectrum Disorder. J Autism Dev Disord.

[60] Elster A, Jarosik J, VanGeest J, Fleming M (2003). Racial and ethnic disparities in health care for adolescents: a systematic review of the literature. Arch Pediatr Adolesc Med.

[61] Mantwill S, Monestel-Umaña S, Schulz PJ (2015). The Relationship between health literacy and health disparities: a systematic review. PLoS One.

[62] Aranguri C, Davidson B, Ramirez R (2006). Patterns of communication through interpreters: a detailed sociolinguistic analysis. J Gen Intern Med.

