# Supplementary material for: Technology Enabled Clinical Care (TECC): Protocol for a Prospective Longitudinal Cohort Study of Smartphone-Augmented Mental Health Treatment
Source: JMIR Res Protoc. 2021 Jan 14;10(1):e23771. doi: 10.2196/23771 (PMC7813563; doi:10.2196/23771)
Supplement: Multimedia Appendix 1 [file resprot_v10i1e23771_app1.pdf]

TECH ENABLED CLINICAL CARE (TECC)

Eight sessions of face-to-face clinical care infused with digital technology. Each session exposes participants to a new clinical skill.

1. SHARED DECISION MAKING
2. IDENTIFYING THOUGHT PATTERNS
3. IDENTIFYING COPING SKILLS AND DEFENSE MECHANISMS
4. COGNITIVE DEFUSION
5. BEHAVIORAL ACTIVATION
6. MINDFULNESS
7. SKILL CARD
8. PATIENT ACTIVATION AND EMPOWERMENT

Digital Clinic:
Acknowledgement of Services

I _________________________ acknowledge and understand the digital information I input into the mindLAMP app will not be monitored in real time by any clinical or staff members of the Digital Clinic.

I _________________________ acknowledge and understand if I experience a clinical emergency, I must independently contact my local hospital or dial 911.

I _________________________ acknowledge and understand services provided by the Digital Clinic do not include emergency services.

I __________________________ acknowledge and understand my data will be reviewed with me during official Digital Clinic meetings.

I _________________________ acknowledge and understand I will use Digital Clinic meetings to ensure I know what data is being collected in the mindLAMP app and how the data is being used in my care.

____
(Print Name)

____ ______________________
(Signature) (Date)

THE DIVISION OF DIGITAL PSYCHIATRY • AT BIDMC

<u>SAFETY PLAN</u>

Please Note: If you are experiencing a medical or psychiatric emergency please contact your local hospital or call 911.

Warning signs

What do you experience when you start to feel extremely depressed or start to think about hurting yourself? List warning signs (thoughts, images, thinking processes, mood, and/or behaviors).

1.
2.
3.

Actions I can take make my environment safer:

1.
2.
3.

Things I can do to distract myself without contacting anyone:

1.
2.
3.

Social situations and people that can help to distract me:

1.
2.
3.

People who I can call/ask for help

1.
2.
3.

Professionals or agencies I can contact during a crisis:

1.
2.
3.

USA National Suicide Hotlines	1-800-SUICIDE (**1-800-784-2433**)<br>1-800-273-TALK (**1-800-273-8255)**
Samaritan's Hotline	**1-877-870-4673**
Boston Emergency Services	**1-800-981-4357**
SafeLink	**1-877-785-2020**
Alcoholics Anonymous	**1-617-426-9444**
Narcotics Anonymous	**1-866-624-3578**
Detox Hotline	**1-800-327-5050**

Shared Decision Making

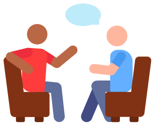

Please list three short-term (6 months or less) goals you would like to work on.

1.
2.
3.

Please list three long-term (6 months or longer) goals you would like to work on.

1.
2.
3.

Please list three ways you could improve your overall health over the course of the next year.

1.
2.
3.

Please list three ways you could improve your mental health over the course of the next year.

1.
2.
3.

Thought patterns and definitions

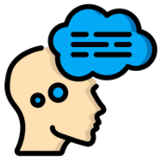

All-or-nothing thinking:
Putting experiences in one of two categories
Examples-- People are all good or all bad.

Overgeneralization:
Believing that something will always happen because it happened once
Example—I will never be able to speak in public because I once had a panic attack before giving a speech.

Discounting the positive:
Deciding that if a good thing happens, it must not be important or doesn't count
Example—I passed the exam this time, but it was a fluke.

Jumping to Conclusions:
Deciding how to respond to a situation without having all the information
Example: The person I am interested in never called me back because they think I am stupid.

Mind Reading:
Believing that you know how someone else is feeling or what they are thinking without any evidence
Example—I know they hate me.

Fortunetelling:
Believing that you can predict a future outcome, while ignoring other alternatives
Example—I am going to fail this test.

Catastrophic Thinking:
Distorting the importance of negative events
Example—I said the wrong thing so now I will never have friends.

Minimizing:
Distorting the importance of positive events
Example—It doesn't matter if I had a good day because I will never be happy.

Emotional Reasoning:
Believing something to be true because it feels true.
Example—I am stupid because I feel stupid

"Should-y" Thinking:
Telling yourself you should, should not, or should have done something when it is more accurate to say you would have preferred or wished you had or had not done something

Thought patterns and definitions

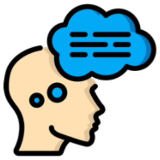

Example—I should be perfect.

Personalization:
Taking blame for some negative event even though you were not responsible, you could not have known to do differently, there were extenuating circumstances, or other people were involved
Example—My childhood was unhappy because of me.

Defense Mechanisms:
Terminology and Definitions

Denial:
Unable to accept external reality

Distortion:
Reshaping external reality to meet internal demands

Projection:
Attribution of one's own undesirable thoughts, impulses, or feelings onto another person

Projection Identification:
Attribution of one's own undesirable thoughts, impulses, or feelings onto another person, and contributing to control it from a distance

Splitting:
People and ideas are separated into "all good" or "all bad."

Regression:
Temporary reversion of the ego to an earlier stage of development

Dissociation:
Separating a painful idea or memory from the feeling attached to it; or a sense of separation of the mind or thinking process from the body

Acting out:
Expression of an unconscious wish is in action

Displacement:
Shifting strong impulses and feelings to safe objects (including people) or situations

Reaction formation:
Converting unconscious thoughts, feelings, and desires perceived to be dangerous onto their opposites.

Repression:
Pushing a desire into the unconscious

Sublimation:
Transforming negative thoughts or feelings into positive, creative ones

Intellectualization:
Isolating the intellectual capacities from emotional experiences

Adapted from: Goldstein, 1984; Goldstein, 1995; Schamess & Shilkret, 2011

Defense Mechanisms:
Terminology and Definitions

Humor:
Making difficult or objectionable experiences funny, including anger and aggression

Withdrawal:
Removing oneself from events or settings that cause distress

Altruism:
Using painful experiences/feelings to motivate self-sacrificing service and participation in causes and events.

Adapted from: Goldstein, 1984; Goldstein, 1995; Schamess & Shilkret, 2011

Identifying Coping Skills

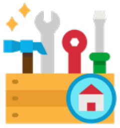

Connecting thoughts, feelings, and behaviors.

I know I am **happy** when I:

Thought: ___________________________________

Feeling: ___________________________________

Behavior: ____________________________________

I know I feel **angry** when I:

Thought: ___________________________________

Feeling: ___________________________________

Behavior: ____________________________________

I know I feel **sad** when I:

Thought: ___________________________________

Feeling: ___________________________________

Behavior: ____________________________________

Identifying Coping Skills

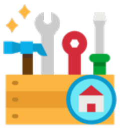

I know I feel **scared** when I:

Thought: ______________________________

Feeling: ______________________________

Behavior: ______________________________

I know I strongly **do not like something** when I:

Thought: ______________________________

Feeling: ______________________________

Behavior: ______________________________

Identifying Coping Skills

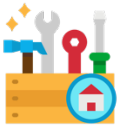

Coping Skills and Behaviors:

When I feel happy I

Behavior: _______________________________

I feel (terrible) (not great) (fine) (good) (great) after I complete this action.

Circle one option

When I feel angry I

Behavior: _______________________________

I feel (terrible) (not great) (fine) (good) (great) after I complete this action.

Circle one option

When I feel sad I

Behavior: _______________________________

I feel (terrible) (not great) (fine) (good) (great) after I complete this action.

Circle one option

When I feel scared I

Behavior: _______________________________

I feel (terrible) (not great) (fine) (good) (great) after I complete this action.

Circle one option

Identifying Coping Skills

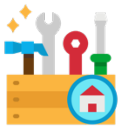

When I strongly do not like something I

Behavior: ________________________________

I feel (terrible) (not great) (fine) (good) (great) after I complete this action.

Circle one option

Behavioral Activation

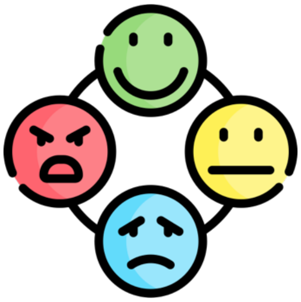

Behavioral Activation:
Goal: To become aware of avoidance behaviors and acknowledge environmental triggers.
Unlike Cognitive Behavior Therapy (CBT), the emphasis is on environmental factors, not thought patterns.

1. List three uncomfortable emotions you had today.
   (Example: I felt **shame** today)

   A.

   B.

   C.

2. List three behaviors or activities you used to decrease the uncomfortable emotions.
   (Example: When I felt shame, I **yelled** at my partner.)

   A.

   B.

   C.

3. List any environmental triggers you noticed while experiencing uncomfortable emotions.
   (Example: I felt shame after being **not getting a promotion** at work.)

   A.

   B.

   C.

Cognitive Defusion

Directions:
For the following exercise, identify a thought, feeling and behavior for the adjectives in bold. Then write a sentence in the third person combining the thought, feeling and behavior. Say the completed sentence out loud.

Example:
I know I am **angry** when I:

Thought: Picture yelling at someone in my mind.

Feeling: Can feel my heart race.

Behavior to decrease feeling: Deep breaths

Third Person Sentence:
Sally knows she is angry when she pictures yelling at someone in her mind. Sally feels angry when her heart begins to race. Sally takes a deep breath when she feels angry.

I know I am **sad** when I:

Thought:

Feeling:

Behavior to decrease feeling:

Third Person Sentence:

I know I am **scared** when I:

Thought:

Feeling:

Behavior to decrease feeling:

Third Person Sentence:

Cognitive Defusion

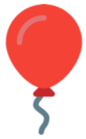

I know I strongly **do not like something** when I:

Thought:

Feeling:

Behavior to decrease feeling:

Third Person Sentence:

I know I am **angry** when I:

Thought:

Feeling:

Behavior to decrease feeling:

Third Person Sentence:

Mindfulness

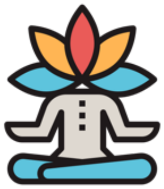

One Minute Mindfulness Exercises

1. Yawn and stretch for 10 seconds every hour.
Do a fake yawn if you have to. That will trigger real ones. Say “ahh” as you exhale. Notice how a yawn interrupts your thoughts and feelings. This brings you into the present. Then stretch really, really slowly for at least 10 seconds. Notice any tightness and say “ease” or just say hello to that place (being mindful — noticing without judgment). Take another 20 seconds to notice and then get back to what you were doing.

2. Three hugs, three big breaths exercise.
Hug someone tight and take 3 big breaths together. Even if they don’t breathe with you, your breathing will ground them.

3. Stroke your hands.
Lower or close your eyes. Take the index finger of your right hand and slowly move it up and down on the outside of your fingers. Once you have mindfully stroked your left hand, swap and let your left-hand stroke the fingers of your right hand.

4. Mindfully eat a raisin.
Take a raisin or a piece of chocolate and mindfully eat it. Slow down, sense it, savor it and smile between bites. Purposefully slow down. Use all your senses to see it, touch it, smell it, and sense it. Then gently pop it into your mouth and really savor it. Savor its texture, its taste, how it feels in your mouth. Let it linger and then swallow it. After you have swallowed it, let your lips turn up slightly and smile. Do the same thing for each raisin you eat or bite you take.

5. Clench your fist and breathe into your fingers.
Position your fingers and thumbs facing down. Now clench your fist tightly. Turn your hand over so your fingers and thumbs are facing up and breathe into your fist. Notice what happens.

6. STOP.
Stand up and breathe. Feel your connection to the earth.
Tune in to your body. Lower your gaze. Scan your body and notice physical sensations or emotions. Discharge any unpleasant sensations, emotions or feelings on the out breath. Notice any pleasant ones and let them fill you up on the in breath.
Observe. Lift your eyes and take in your surroundings. Observe something in your environment that is pleasant and be grateful for it and its beauty.

Created by: Leonie Stewart-Weeks March 30, 2020
https://psychcentral.com/blog/1-minute-mindfulness-exercises/

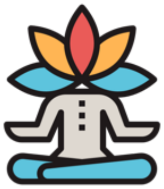

Possibility. Ask yourself what is possible or what is new or what is a forward step.
If you find yourself being reactive, try the following steps:

- Pause and take one to three big breaths.
- Say “step back.” (You don’t have to physically step back, you can just do it in your mind.)
- Say “clear head.”
- Say “calm body.”
- Breathe again. Say “relax,” “melt” or “ease.”

7. Mindful breathing for one minute.

Lower your eyes and notice where you feel your breath. That might be the air going in and out at your nostrils or the rise and fall of your chest or stomach. If you can’t feel anything, place your hand on your stomach and notice how your hand gently rises and falls with your breath. If you like, you can just lengthen the in breath and the out breath or just breathe naturally. Your body knows how to breathe. Focus on your breath. When your mind wanders, as it will do, just bring your attention back to your breath. You might like to say ‘thinking’ when you notice your thoughts and just gently shepherd your attention back to your breath. This can be done for longer than one minute. However, even for one minute it will allow you to pause and be in the moment. Or you might just like to breathe out stress on the out breath and breathe in peace on the in breath.

8. Loving-kindness meditation.
For one minute, repeat ‘May I be happy, may I be well, may I be filled with kindness and peace.’ You can substitute “you” for “I” and think of someone you know and like, or just send love to all people.

9. An aspiration.
Decide on an aspiration. Just ask yourself this question: What is my heart’s aspiration? Pause for about 20 seconds. Do this a second or third time and write down what comes. Perhaps it is to come from love, or to be kind to yourself or others or to be patient. Once you decide which aspiration you like best, say that at the beginning of the day. This will set you up for your day and your interactions with others (and even with yourself).

Skill Card

Instructions:
Identify the skill(s) you practiced today and write three sentences describing your experience.

Shared Decision Making 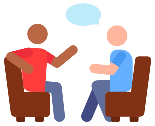
Identifying Thought Patterns 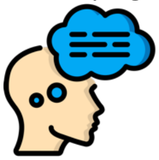
Identifying Coping Skills 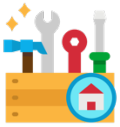

Skill Card

Identifying Defense Mechanisms:

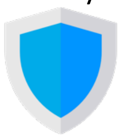

Cognitive Defusion

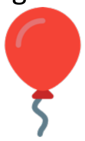

Behavioral Activation

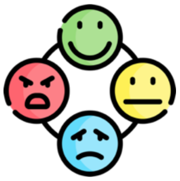

Skill Card

Mindfulness 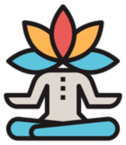
Patient Activation & Empowerment 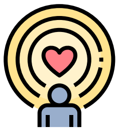

Patient Activation & Empowerment

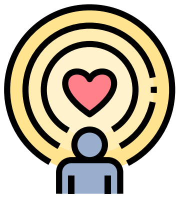

Identify three goals you have related to your general health care needs.

1.
2.
3.

Identify three coping skills/ strengths that can help you achieve the above goals.

1.
2.
3.

Identify three potential barriers to achieving your above goals.

1.
2.
3.

Identify three ways a health care professional may be able to assist you in achieving your goals.

1.
2.
3.
